# Effect of sarpogrelate and high‐dose statin on the reduction of coronary spasm in vasospastic angina: A two by two factorial, pilot randomized study

**DOI:** 10.1002/clc.23239

**Published:** 2019-07-24

**Authors:** So Ree Kim, Ki Hong Choi, Young Bin Song, Joo Myung Lee, Taek Kyu Park, Jeong Hoon Yang, Joo‐Yong Hahn, Jin‐Ho Choi, Seung‐Hyuk Choi, Hyeon‐Cheol Gwon

**Affiliations:** ^1^ Division of Cardiology, Department of Internal Medicine, Heart Vascular Stroke Institute, Samsung Medical Center Sungkyunkwan University School of Medicine Seoul Republic of Korea; ^2^ Department of Critical Care Medicine, Samsung Medical Center Sungkyunkwan University School of Medicine Seoul Republic of Korea; ^3^ Department of Emergency Medicine, Samsung Medical Center Sungkyunkwan University School of Medicine Seoul Republic of Korea

**Keywords:** coronary spasm, high‐dose statin, remission, sarpogrelate, vasospastic angina

## Abstract

**Background:**

Vasospastic angina (VSA) is characterized by coronary spasm, which can be aggravated by vasoactive substances such as serotonin. Hypothesis Sarpogrelate, a selective serotonin receptor antagonist, and high‐dose statin have some effects on the reduction of coronary spasm in patients with VSA.

**Methods:**

We recruited 100 patients with angiographically confirmed VSA, and randomly assigned them into four groups: sarpogrelate with high‐dose statin (Group A, n = 25), sarpogrelate with low‐dose or no statin (Group B, n = 25), placebo with high‐dose statin (Group C, n = 25), and placebo with low‐dose or no statin (Group D, n = 25). The primary endpoint was the remission of coronary spasm on 1‐year follow‐up provocation test.

**Results:**

The most common site of coronary spasm was left anterior descending artery (42%). Most patients (96%) took calcium channel blockers, and 46% were treated with vasodilators. Overall, 40% of patients reported no chest pain at 1 year, and 23% showed complete remission of coronary spasm on 1‐year follow‐up provocation test. No difference was observed in symptomatic and angiographically complete remission rate between the sarpogrelate and the placebo group. Although the apolipoprotein B level at the 1‐year follow‐up was significantly lower in the high‐dose statin group, symptomatic and angiographic outcomes were not different according to statin intensity. Distal thrombolysis in myocardial infarction (TIMI) flow on initial provocation test was independently associated with angiographically complete remission.

**Conclusions:**

Sarpogrelate or high‐dose statin did not significantly improve the angiographic remission rate in patients with VSA. Distal TIMI flow on initial provocation test could predict the complete remission of coronary spasm at follow‐up.

**Abbreviations**
CAG: coronary angiography
CCB: calcium channel blocker
CI: confidence interval
OR: odds ratio
TIMI: thrombolysis in myocardial infarction
VSA: vasospastic angina

## 1 INTRODUCTION

Vasospastic angina (VSA) is a clinical syndrome characterized by transient ST‐segment elevation with recurrent episodes of angina at rest, due to spontaneous coronary artery spasm leading to myocardial ischemia.^1^, ^2^ Ischemic episodes of VSA are aggravated by physical or psychological stress, exposure to the cold, or hyperventilation.^3^, ^4^, ^5^ Furthermore, several pharmacologic agents such as catecholamines, para‐sympathomimetic agents, anticholinesterase agents, beta‐adrenergic blocking agents, ergonovine, serotonin, and histamine can induce coronary spasm.^6^, ^7^, ^8^, ^9^ Serotonin (5‐hydroxytryptamine or 5‐HT) mediates vasoconstriction and platelet aggregation by 5‐HT_2A_ receptors on vascular smooth muscle cells and platelets. Sarpogrelate is a selective 5‐HT_2A_ antagonist, which inhibits vasoconstriction and platelet aggregation, and is theoretically beneficial in the treatment of VSA.^10^ However, there are no data on treating patients with VSA, even though sarpogrelate has been widely used as an antiplatelet agent in peripheral artery disease.

Although the pathogenesis of VSA is not yet fully understood, vascular endothelial dysfunction, coronary microvascular dysfunction, and Ca^2+^‐mediated vascular smooth muscle cell hyperplasia have been suggested to be the main contributors.^11^, ^12^, ^13^, ^14^ Moreover, some investigators have proposed that coronary spasm is an early form of atherosclerosis, based on the results of several animal studies and intravascular imaging studies for assessment of spastic artery.^15^, ^16^, ^17^, ^18^ However, there has been debate regarding the beneficial effects of statin, a well‐known coronary artery plaque stabilizer, for patients with VSA.^19^, ^20^

To address this debate, we conducted a 2 × 2 factorial randomized controlled trial to evaluate the efficacy of sarpogrelate and high‐dose statin for reduction of coronary spasm in patients with VSA.

## 2 METHODS

### 2.1 Study design and population

The present study was a 2 × 2 randomized prospective single‐center trial. The study protocol was approved by the Institutional Review Board of Samsung Medical Center, and all patients provided written informed consent. From 2012 to 2016, 20‐ to 70‐year‐old patients with VSA were recruited. At the initial evaluation, coronary spasm was angiographically proven, which was defined as thrombolysis in myocardial infarction (TIMI) flow of less than 3 with chest pain or significant ST‐T wave change by spontaneous coronary spasm or intracoronary ergonovine spasm provocation test. The main exclusion criteria were cardiac arrest due to coronary spasm, left main spasm, significant fixed coronary artery stenosis of more than 70%, severe left ventricular dysfunction (ejection fraction <30%), bleeding tendency, and significant liver and kidney disease. Patients with coagulation disorders, platelet count less than 50 000/μL, or prothrombin time more than 2.0 (international normalized ratio) were considered to indicate bleeding tendency. Significant liver disease was defined as aspartate aminotransferase or alanine aminotransferase of more than 100 U/mL, and renal failure was defined as serum creatinine of more than 2.0 mg/dL. Pregnant or breastfeeding patients were also excluded. Clinical Trial Registration Information: ClinicalTrials.gov, NCT01674686.

A total of 100 VSA patients were included. They were randomly assigned to a sarpogrelate or a placebo group at a one‐to‐one ratio. Each group was also randomly divided into a high‐dose statin and a low‐dose or no statin group. Randomization was done via a web‐based system by computer‐generated block randomization. The dose of sarpogrelate was 100 mg twice a day. The high‐dose statin group was prescribed atorvastatin 80 mg once a day, while the low‐dose or no statin group was prescribed simvastatin 20 mg once a day or no statin medication. Group A took sarpogrelate and high‐dose statin, group B took sarpogrelate and low‐dose or no statin, group C took placebo and high‐dose statin, and group D took placebo and low‐dose or no statin. Each group had 25 patients (Supporting Information, Figure S1). After randomization, to control symptoms, use of calcium channel blockers (CCBs) or nitrates was based on physician discretion.

### 2.2 Study procedures and follow‐up

Before the spasm provocation test, routine right and left coronary angiography (CAG) was performed. If any significant stenosis (angiographic luminal stenosis >70%) was observed, the spasm provocation test was canceled. After performing routine CAG, the intracoronary ergonovine spasm provocation test was started using the left coronary artery first. Two bolus doses of ergonovine maleate were injected into each coronary artery at an interval of 2 minutes: 10 μg followed by 20 μg for the left coronary artery and 10 μg followed by another 10 μg for the right. Before injection of the second dose, CAG was performed and 12‐lead ECG was taken. The test was stopped immediately after a positive result was obtained, and intracoronary nitroglycerin 200 to 600 mg was administered until the spasm had been relieved angiographically.

Vital signs, height, body weight, and blood tests including complete blood count, lipid profile, liver function test, blood urea nitrogen, creatinine, and C‐reactive protein were checked at baseline, 1, 3, 6, and 12 months of follow‐up. Echocardiography was performed at baseline. Prothrombin time level was checked at baseline, and creatine kinase level was checked at 1 and 12 months of follow‐up. Apolipoprotein B, apolipoprotein A1, and lipoprotein (a) levels were checked at baseline and 12 months of follow‐up.

### 2.3 Outcomes

Coronary arteries with luminal diameters of more than 2.5 mm were included for analysis of angiographic findings. The primary outcome was remission of coronary spasm at the 1‐year follow‐up CAG with spasm provocation test. Vasodilators or CCBs were held 48 hours before the spasm provocation test. Remission of coronary spasm was defined as improvement in distal TIMI flow grade in the spastic vessel compared to the baseline provocation test. Complete remission was defined as disappearance of coronary artery spasm at the 1‐year follow‐up provocation test. If one vessel showed improvement while the other vessel showed aggravation of coronary spasm, the angiographic result was considered to be “no interval change.” Secondary outcomes were changes of lipid profile, apolipoprotein B, apolipoprotein A1, and lipoprotein (a) levels over 1 year.

### 2.4 Statistical analysis

Continuous variables were compared using the Student's *t*‐test or Mann‐Whitney test where applicable. Categorical data were assessed using the chi‐square test or Fisher's exact test, as appropriate. To identify independent predictors of complete remission, the odds ratio (OR) and 95% confidence interval (CI) were calculated using a multivariate logistic regression model that included sex, hypertension, diabetes mellitus, alcohol abstinence, sarpogrelate, high‐dose statin, and distal TIMI flow 0 at the initial provocation test. Statistical analyses were performed with SPSS software version 18 (IBM Corp, Armonk, New York). All tests were two‐tailed and *P* < .05 was considered statistically significant.

## 3 RESULTS

### 3.1 Baseline clinical and angiographic characteristics according to sarpogrelate

Baseline clinical and angiographic characteristics in the sarpogrelate group vs the placebo group are shown in Table 1. Overall, the mean age was 57.4 years, 92% were men, and 78% drank alcohol. Most patients (96.0%) had been prescribed CCBs, and 46% had been prescribed vasodilators after angiographically proven vasospasm. There were no significant differences in general cardiovascular risk factors, alcohol intake, results of laboratory tests, and medication at discharge between the sarpogrelate and placebo groups (Table 1).

**Table 1:** Baseline clinical and angiographic characteristics in the sarpogrelate vs the placebo groups

	Total (n = 100)	Sarpogrelate (n = 50)	Placebo (n = 50)	*P*‐value
Age (y)	57.4 ± 7.8	57.6 ± 7.7	57.3 ± 7.9	0.850
Male	92 (92)	48 (96)	44 (88)	0.269
Systolic blood pressure (mm Hg)	122.8 ± 17.2	121.1 ± 18.0	124.5 ± 16.4	0.335
Diabetes mellitus	10 (10.0)	7 (14.0)	3 (6.0)	0.182
Hypertension	34 (34.0)	14 (28.0)	20 (40.0)	0.205
Dyslipidemia	14 (14.0)	6 (12.0)	8 (16.0)	0.564
History of PCI	4 (4.0)	1 (2.0)	3 (6.0)	0.617
Alcohol	78 (78.0)	41 (82.0)	37 (74.0)	0.334
Current smoker	39 (39.0)	19 (38.0)	20 (40.0)	0.366
Laboratory tests				
Total cholesterol (mg/dL)	172.2 ± 42.1	171.3 ± 38.3	173.2 ± 46.0	0.819
Triglyceride (mg/dL)	124 (24‐764)	130 (24‐764)	115 (30‐514)	0.391
HDL (mg/dL)	49 (24‐145)	49 (24‐101)	48 (29‐145)	0.576
LDL (mg/dL)	103.2 ± 35.4	104.1 ± 34.5	102.4 ± 36.6	0.805
Creatinine (mg/dL)	0.91 ± 0.16	0.91 ± 0.17	0.91 ± 0.16	0.966
CRP (mg/dL)	0.06 (0.03‐3.14)	0.06 (0.03‐3.14)	0.06 (0.03‐1.50)	0.582
Apolipoprotein B (mg/dL)	78.3 ± 21.9	76.4 ± 19.0	80.0 ± 24.4	0.418
Apolipoprotein A1 (mg/dL)	116.0 ± 27.9	118.1 ± 26.7	114.1 ± 29.1	0.489
Lipoprotein(a) (mg/dL)	15.0 ± 13.1	11.7 (1.8‐57.8)	10.8 (0.4‐46.8)	0.760
Medication				
Calcium channel blocker, n (%)	96 (96.0)	46 (92.0)	50 (100.0)	0.117
Vasodilators	46 (46.0)	25 (50.0)	21 (42.0)	0.422
Coronary angiography				
Coronary stenosis^a^				
No fixed stenosis	47 (47.0)	25 (50.0)	22 (44.0)	0.548
LAD	36 (36.0)	18 (36.0)	18 (36.0)	1.000
LAD %DS	30 (20‐60)	30 (20–60)	30 (20‐50)	0.990
LCX	19 (19.0)	9 (18.0)	10 (20.0)	0.799
LCX %DS	30 (20–60)	30 (20‐60)	40 (30‐60)	0.586
RCA	12 (12.0)	4 (8.0)	8 (16.0)	0.218
RCA %DS	30 (20–60)	35 (20‐60)	30 (20‐40)	0.246
Spasm location				0.246
Multivessel	2 (2.0)	0 (0)	2 (4.0)	
LAD	42 (42.0)	25 (50.0)	17 (34.0)	
LCX	21 (21.0)	10 (20.0)	11 (22.0)	
RCA	35 (35.0)	15 (30.0)	20 (40.0)	
Spontaneous spasm	3 (3.0)	1 (2.0)	2 (4.0)	1.000
Distal TIMI flow				0.840
TIMI 0	57 (57.0)	28 (56.0)	29 (58.0)	
TIMI ≥1	43 (43.0)	22 (44.0)	21 (42.0)	

Abbreviations: CRP, C‐reactive protein; HDL, high‐density lipoprotein; LAD, left anterior descending artery; LCX, left circumflex artery; LDL, low‐density lipoprotein; %DS, percent diameter stenosis; PCI, percutaneous coronary intervention; RCA, right coronary artery; TIMI, thrombolysis in myocardial infarction.

Data are presented as mean ± SD, median (range), or n (%).

a Fixed stenosis was observed in 53 patients and 67 vessels were involved.

Forty‐seven patients showed no fixed stenosis on initial CAG. Fixed stenosis was observed in 53 patients and 67 vessels were involved. Thirty‐six patients had fixed stenosis at left anterior descending artery (LAD) (median percent diameter stenosis [%DS], 30% [range from 20 to 60]), 19 at left circumflex artery (LCX) (median %DS, 30% [range from 20 to 60]), and 12 at right coronary artery (median %DS, 30% [range from 20 to 60]). There was no significant difference of fixed stenosis severity between the sarpogrelate group and the placebo group (Table 1). Coronary vasospasm occurred at the site with fixed stenosis in 44 patients.

Most of the patients showed transient luminal narrowing of an epicardial vessel of 90% or greater. Only one patient showed 75% of transient luminal narrowing with angina and ischemic ECG changes. No patient was diagnosed with microvascular spasm, which was defined as angina symptom with ischemic ECG changes without significant epicardial coronary artery spasm (>75%).^21^ There were two patients with multivessel spasm involving the LAD and the LCX at the same time. Spontaneous spasm was observed in three patients. Forty‐two percent of the coronary spasms were located at LAD, which was followed by the right coronary artery at 35% and LCX at 21%. Fifty‐seven percent of the patients showed distal TIMI 0 flow after intracoronary ergonovine injection. There were no differences in spasm location and grade of distal TIMI flow between the sarpogrelate and placebo groups (Table 1).

### 3.2 Baseline clinical and angiographic characteristics according to statin intensity

Baseline clinical and angiographic characteristics according to statin therapy are shown in Table S1. The proportion of cardiovascular risk factors, alcohol intake, blood tests including lipid profiles, medication at discharge, location and grade of fixed stenosis, spasm location, and grade of distal TIMI flow were similar between the high‐dose statin group and the low‐dose or no statin group, with the exception of age. Patients in the high‐dose statin group were older than those in the low‐dose or no statin group.

### 3.3 Outcomes

No specific complications occurred during provocation test at baseline and follow‐up in this study. Each group showed good compliance with both drugs (sarpogrelate vs placebo, and high‐dose statin vs low‐dose or no statin), and there was no statistically significant difference among the four groups (Figure S2). In the four‐group comparison, the rate of remission (Group A 47.4%, Group B 58.3%, Group C 40.0%, and Group D 56.3%, *P* = .744) and the rate of performing follow‐up angiography at 1‐year (Group A 30.6%, Group B 19.4%, Group C 24.2%, and Group D 25.8%, *P* = .236) was not different.

Table 2 presents the primary and secondary outcomes according to the sarpogrelate administration. A total of 65 patients visited the outpatient clinic 1 year after the index procedure, and 62 patients underwent CAG with spasm provocation test at the 1‐year follow‐up. Overall, 40% of patients reported no chest pain at the 1‐year clinical follow‐up. In 62 angiographically followed patients, no difference was observed in progression or improvement of fixed stenosis between the sarpogrelate and the placebo groups. In regard to coronary vasospasm, 55% (n = 34) showed improved distal TIMI flow in the spastic vessel compared with the baseline spasm provocation test, and 23% (n = 14) showed complete remission of coronary vasospasm, despite stopping of CCBs or vasodilators 48 hours before the follow‐up provocation test. However, the proportion of total remission (sarpogrelate vs placebo, 51.6% vs 58.1%, *P* = .610) and complete remission (25.8% vs 19.4%, *P* = .544) did not differ between the two groups. Similarly, there were no significant differences in the secondary outcomes, including the values of lipid profiles, apolipoprotein B, apolipoprotein A1, or lipoprotein (a) at the 1‐year follow‐up between the sarpogrelate and placebo groups.

**Table 2:** Outcomes in sarpogrelate vs placebo group

1‐year follow‐up^a^	Sarpogrelate (n = 31)	Placebo (n = 34)	*P*‐value
Symptom‐free	12 (38.7)	14 (41.2)	0.839
Alcohol abstinence	10 (32.3)	13 (41.9)	0.430
Follow‐up CAG^b^ (N = 62)	N = 31	N = 31	
Coronary stenosis			
No change	24 (77.4)	27 (87.1)	0.319
Improvement	3 (9.7)	2 (6.5)	1.000
Progression	4 (12.9)	2 (6.5)	0.671
PCI	1 (3.2)	1 (3.2)	1.000
Coronary vasospasm			
No change	15 (48.4)	13 (41.9)	0.610
Remission	16 (51.6)	18 (58.1)	0.610
Complete remission	8 (25.8)	6 (19.4)	0.544
Progression	0 (0)	0 (0)	
Total cholesterol (mg/dL)	150.1 ± 35.6	143.7 ± 34.6	0.481
Triglyceride (mg/dL)	112.4 ± 50.3	104.5 ± 52.0	0.549
HDL (mg/dL)	53.8 ± 13.2	55.7 ± 13.9	0.586
LDL (mg/dL)	84.1 ± 33.1	77.4 ± 29.5	0.415
Apolipoprotein B (mg/dL)	69.7 ± 22.6	66.2 ± 19.4	0.530
Apolipoprotein A1 (mg/dL)	133.6 ± 28.7	130.1 ± 26.2	0.639
Lipoprotein(a) (mg/dL)	10.8 (1.9‐47.9)	12.4 (1.3‐79.4)	0.346

Abbreviations: CAG, coronary angiography; HDL, high‐density lipoprotein; LDL, low‐density lipoprotein; PCI, percutaneous coronary intervention.

Data are presented as mean ± SD, median (range), or n (%).

a 65 patients visited the outpatient clinic for 1‐year follow‐up.

b 62 patients underwent CAG for 1‐year follow‐up.

**Figure 1:**
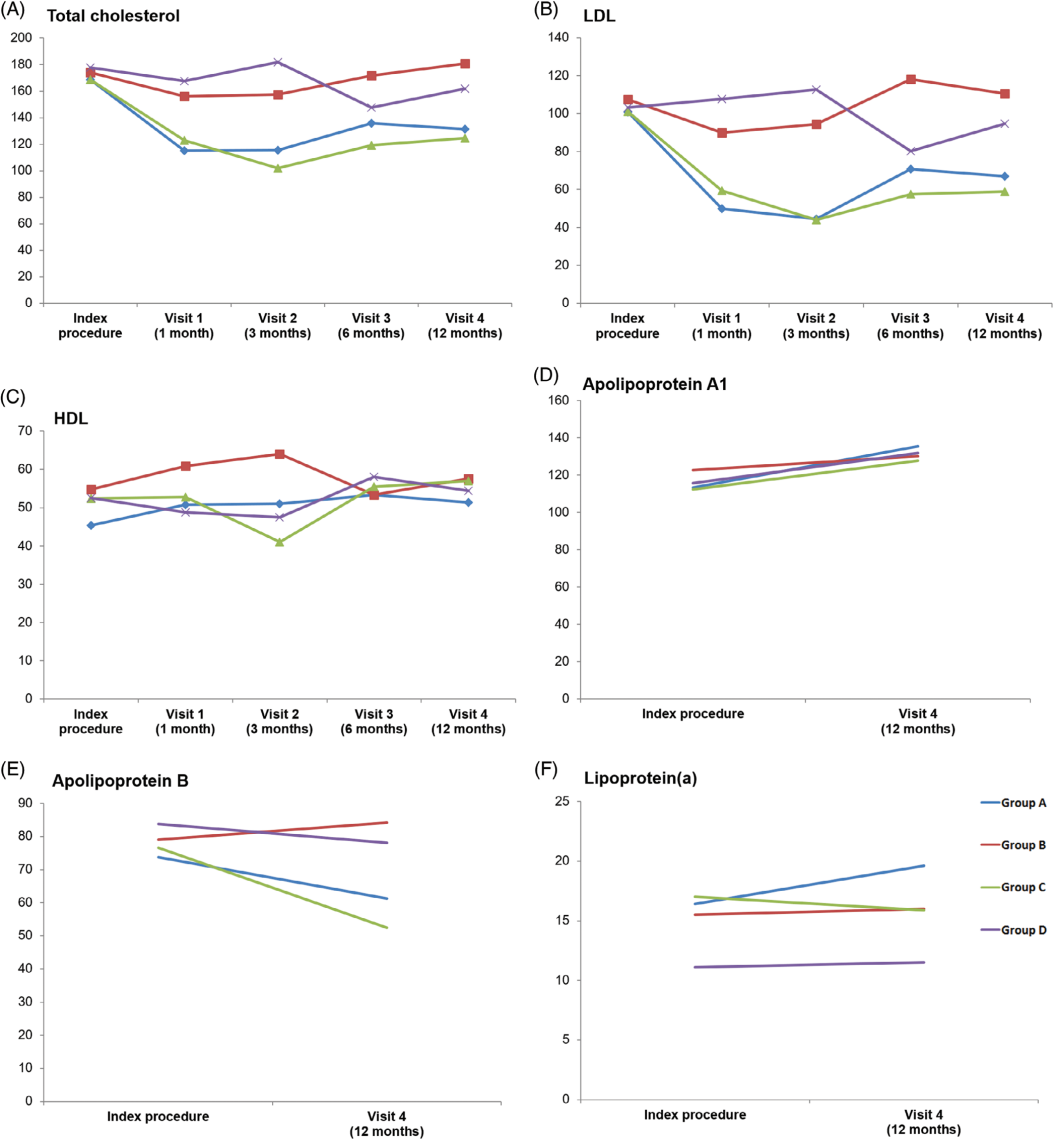
Trends of lipid profile. Curves denote trends of lipid profile in each group over 12 months of follow‐up. Group A, sarpogrelate with high‐dose statin group; Group B, sarpogrelate with low‐dose or no statin group; Group C, placebo with high‐dose statin group; Group D, placebo with low‐dose or no statin group. Abbreviations: HDL, high‐density lipoprotein; LDL, low‐density lipoprotein

Outcomes according to statin therapy are presented in Table 3. More patients showed improvement of fixed stenosis in the high dose statin group than in the low‐dose or no statin group without statistical significance (4 vs 1, *P* = .366). The alcohol abstinence rate was not different between the two groups. Neither the symptomatic remission nor the angiographically complete remission rates were different between the high‐dose statin and the low‐dose or no statin groups. Triglyceride, apolipoprotein A1, and lipoprotein (a) levels were not different according to statin therapy. On the other hand, the total cholesterol, low‐density lipoprotein (LDL), and apolipoprotein B levels were significantly lower in the high‐dose statin group than in the low‐dose or no statin group (Figure 1).

**Table 3:** Outcomes according to statin therapy

1‐year follow‐up^a^	High‐dose statin (n = 36)	Low‐dose or no statin (n = 29)	*P*‐value
Symptom‐free	21 (58.3)	18 (62.1)	0.760
Alcohol abstinence	13 (38.2)	10 (35.7)	0.838
Follow‐up CAG^b^ (N = 62)	N = 34	N = 28	
Coronary stenosis			
No change	27 (79.4)	24 (85.7)	0.740
Improvement	4 (11.8)	1 (3.6)	0.366
Progression	3 (8.8)	3 (10.7)	1.000
PCI	2 (5.9)	0 (0)	0.497
Coronary vasospasm			
No change	16 (47.1)	12 (42.9)	0.741
Remission	18 (52.9)	16 (57.1)	0.741
Complete remission	6 (17.6)	8 (28.6)	0.306
Progression	0 (0)	0 (0)	
Total cholesterol (mg/dL)	128.2 ± 24.2	169.4 ± 32.9	<0.001
Triglyceride (mg/dL)	99.5 ± 43.6	119.0 ± 57.6	0.141
HDL (mg/dL)	54.0 ± 10.9	55.7 ± 16.3	0.638
LDL (mg/dL)	63.2 ± 20.7	101.2 ± 29.0	<0.001
Apolipoprotein B (mg/dL)	57.5 ± 17.6	80.5 ± 17.5	<0.001
Apolipoprotein A1 (mg/dL)	132.3 ± 21.5	131.3 ± 33.4	0.895
Lipoprotein (a) (mg/dL)	15.5 (1.3‐79.4)	9.9 (1.9‐44.2)	0.185

Abbreviations: CAG, coronary angiography; HDL, high‐density lipoprotein; LDL, low‐density lipoprotein; PCI, percutaneous coronary intervention.

Data are presented as mean ± SD, median (range), or n (%).

a 65 patients visited the outpatient clinic for 1‐year follow‐up.

b 62 patients underwent CAG for 1‐year follow‐up.

### 3.4 Independent predictors of angiographically complete remission at the 1‐year follow‐up visit

According to the baseline characteristics of coronary vasospasm, patients were divided into three groups; spontaneous spasm (n = 3), Distal TIMI 0 flow (n = 56), and TIMI 1 or 2 flow (n = 41). Patients who showed transient luminal narrowing with distal TIMI 1 or 2 flow showed higher rate of complete remission of coronary vasospasm at 1‐year compared to others (spontaneous spasm vs distal TIMI 0 vs distal TIMI 1 or 2, 0% vs 15.8% vs 38.1%, *P* = .095) (Table S2). Independent predictors of complete remission were evaluated using a multivariate logistic regression model (Table 4). Sarpogrelate, high‐dose statin, and alcohol abstinence were not associated with angiographically complete remission of VSA. Only distal TIMI 0 flow at initial provocation test was independently associated with lower rates of complete remission on 1‐year follow‐up provocation test (OR 0.23, 95% CI 0.06‐0.93, *P* < .039).

**Table 4:** Independent predictors of angiographically complete remission

Variables	Odds ratio (95% CI)	*P*‐value
Sex	0.73 (0.08‐6.58)	0.779
Hypertension	1.02 (0.23‐4.46)	0.977
Diabetes mellitus	4.50 (0.62‐32.7)	0.138
Alcohol abstinence on 1‐year follow‐up	1.08 (0.26‐4.44)	0.913
Sarpogrelate	1.21 (0.30‐4.82)	0.788
High‐dose statin	0.32 (0.08‐1.31)	0.112
Distal TIMI 0 flow on initial provocation test	0.23 (0.06‐0.93)	0.039

Abbreviations: CAG, coronary angiography; CI, confidence interval; TIMI, thrombolysis in myocardial infarction.

## 4 DISCUSSION

This prospective randomized trial evaluated the efficacy and safety of sarpogrelate and high‐dose statin for patients with VSA in addition to standard treatment including CCB and/or vasodilators. The principal findings of the current study are as follows. First, sarpogrelate did not appear to affect complete remission of coronary vasospasm in VSA. Second, high‐dose statin was attributed to improvement in the lipid profile, but it could not modify the symptomatic and angiographic outcomes in VSA. Finally, distal TIMI 0 flow at the initial provocation test appeared to adversely affect complete remission of coronary spasm on 1‐year follow‐up.

VSA is caused by coronary vasospasm. However, the mechanisms of coronary vasospasm are poorly understood.^22^ Endothelial dysfunction and hyper‐reactivity of vascular smooth muscle cells have been proposed to be the main mechanisms of coronary spasm.^1^, ^23^ CCB and nitrates had been evaluated for decreasing angina symptoms in VSA based on these mechanisms,^24^, ^25^, ^26^ and are recommended in current guidelines to manage VSA.^27^, ^28^ However, some patients have recurrent angina symptoms even while taking sufficient medication including CCB and nitrates. Furthermore, one study also suggested that angina symptoms could occur more frequently during the CCB withdrawal period.^24^

In regard to these limitations, several medications that were perceived to be relevant to coronary spasm were evaluated for managing VSA. Serotonin (5‐hydroxytryptamine, 5‐HT) is one of the most important vasoconstrictor triggers with a possible role in coronary spasm. It mediates vasoconstriction by 5‐HT_2A_ receptors on vascular smooth muscle cells, and also promotes platelet aggregation.^10^ However, the role of serotonin in VSA is controversial.^29^, ^30^, ^31^ Sarpogrelate hydrochloride, a selective 5‐HT_2A_ antagonist, suppresses platelet aggregation and inhibits thrombus formation and vascular smooth muscle cell proliferation, and is widely used to improve vascular function in peripheral arterial disease.^10^ It has been also reported to inhibit serotonin‐induced coronary vasospasm in an animal study^32^ and to improve exercise capacity by increasing collateral flow in effort angina.^33^ On the basis of the known clinical efficacy of sarpogrelate, we hypothesized this medication might have a benefit on VSA. However, no significant effect on VSA was observed with sarpogrelate in the present study.

Previous studies evaluating the morphological characteristics in the spastic vessel using intravascular ultrasound or optical coherence tomography have demonstrated that coronary vasospasm occurs at the site with greater plaque accumulation.^15^, ^34^ As several studies have suggested that 3‐hydroxy‐3‐methylglutaryl‐coenzyme reductase inhibitors (statins) could improve endothelial dysfunction and vascular inflammation,^35^, ^36^ the role of statin therapy in VSA has come to the fore. Yasue et al reported that addition of fluvastatin to a CCB reduced coronary vasospasm at a 6‐month follow‐up provocation test.^37^ In addition, a study from Korea Acute Myocardial Infarction Registry suggested that statin therapy could reduce the 1‐year incidence of myocardial infarction in patients with coronary spasm‐induced AMI.^38^ On the other hand, recent large‐scale studies showed that statin therapy could not reduce the adverse cardiac events in VSA patients.^20^, ^39^ Our data suggest that statin therapy does not modify the VSA‐related symptomatic and angiographic outcomes of VSA patients even though lipid profiles improved over the follow‐up period.

Pathophysiology of VSA is complex. Other than aforementioned mechanisms, inflammation of coronary adventitia and perivascular adipose tissue had been proposed to be associated with coronary vasospasm.^40^ In this regard, anti‐inflammatory therapies might have a role in VSA as shown in myocardial infarction with reducing recurrent cardiovascular event.^41^ Serotonin‐induced coronary vasospasm is only one of many potential pathways that might produce VSA. Large sample‐sized study is needed with medications involving mechanisms of coronary vasospasm. The present study is the first one to perform the 1‐year follow‐up provocation test in patients with VSA and show the possibility of complete remission of VSA. In the present study, the reduction of coronary vasospasm occurred in more than half of the patients with VSA and disappearance of coronary vasospasm in 22% with proper medical management. Furthermore, we found that the severe spasm such as TIMI 0 flow after ergonovine injection was associated with lower rates of complete remission at the 1‐year follow‐up. This result means that patients with more severe spasm at the initial provocation test would have a lower chance of complete remission of coronary vasospasm despite adequate medical management. Therefore, closer follow‐up and intensive titration of medication should be required for VSA patients with severe vasospastic nature at the initial spasm provocation test.

This study has several limitations. First, the sample size and follow‐up rate were low. Based on the results of this pilot study (complete remission rate 20% in the sarpogrelate group and 25% in the placebo group), a total of 2432 patients will be needed to prove the difference between serotonin antagonist and placebo with consideration of 10% withdrawal rates. Due to the invasiveness of the provocation test, the rate of consent withdrawal was high, and only 62% of patients underwent the 1‐year follow‐up spasm test. Second, the selection of treatment strategies other than sarpogrelate and statin was based on the doctor's preference. Third, because most of the enrolled patients were men in this study, evaluation of coronary microvascular spasm, which is known to be more prevalent in women, could not be performed. Finally, we determined the extent of stenosis via visual estimation instead of intravascular ultrasound or optical coherence tomography, and thus stenosis in fixed disease could have been under‐ or overestimated.

## 5 CONCLUSIONS

Sarpogrelate and high‐dose statin did not appear to affect the symptomatic or angiographic outcomes of VSA. With proper medical therapies such as CCB, 23% of followed patients with VSA showed complete remission of coronary spasm at the 1‐year follow‐up. If coronary spasm was severe enough to show distal TIMI 0 flow at the initial spasm test, the chance of complete remission of coronary spasm was lower at the 1‐year follow‐up.

## CONFLICT OF INTEREST

The authors declare no potential conflict of interests.

## Supporting information


**Figure S1.** Study flow
**Figure S2.** Compliance
**Table S1.** Baseline clinical and angiographic characteristics according to statin therapy
**Table S2.** Outcomes according to the characteristics of baseline coronary spasm
