# Supplementary material for: Effect of sarpogrelate and high‐dose statin on the reduction of coronary spasm in vasospastic angina: A two by two factorial, pilot randomized study
Source: Clin Cardiol. 2019 Jul 24;42(10):899–907. doi: 10.1002/clc.23239 (PMC6788571; doi:10.1002/clc.23239)
Supplement: Supplementary file 1 — Figure S1. Study flow Figure S2. Compliance Table S1. Baseline clinical and angiographic characteristics according to statin therapy Table S2. Outcomes according to the characteristics of baseline coronary spasm [file CLC-42-899-s001.docx]

**Effect of sarpogrelate and high-dose statin on the reduction of coronary spasm in vasospastic angina: a 2 by 2 factorial, pilot randomized study**

So Ree Kim, MD^1✝^, Ki Hong Choi, MD^1✝^, Young Bin Song, MD, PhD^1*^, Joo Myung Lee, MD, MPH, PhD^1^, Taek Kyu Park, MD, PhD^1^, Jeong Hoon Yang, MD, PhD^1,2^, Joo-Yong Hahn, MD, PhD^1^, Jin-Ho Choi, MD, PhD^1,3^, Seung-Hyuk Choi, MD, PhD^1^, Hyeon-Cheol Gwon, MD, PhD^1^

**Table of Contents**

**(1) Supplementary Tables**

**(2) Supplementary Figure and Figure Legend**

**(1) Supplementary Tables**

**Supplementary Table 1. Baseline Clinical and Angiographic Characteristics according to Statin Therapy**

|  | **High-dose statin (n = 50)** | **Low-dose or no statin (n = 50)** | **P value** |
| --- | --- | --- | --- |
| Age (years) | 59.4 ± 7.5 | 55.5 ± 7.7 | 0.014 |
| Male | 46 (92.0) | 46 (92.0) | 1.000 |
| Systolic blood pressure (mmHg) | 124.5 ± 19.1 | 121.1 ± 15.1 | 0.329 |
| Diabetes mellitus | 5 (10.0) | 5 (10.0) | 1.000 |
| Hypertension | 16 (32.0) | 18 (36.0) | 0.673 |
| Dyslipidemia | 10 (20.0) | 4 (8.0) | 0.084 |
| History of PCI | 2 (4.0) | 2 (4.0) | 1.000 |
| Alcohol | 38 (76.0) | 40 (80.0) | 0.629 |
| Current smoker | 20 (40.0) | 19 (38.0) | 0.912 |
| Laboratory tests |  |  |  |
| Total cholesterol (mg/dl) | 168.6 ± 43.8 | 175.8 ± 40.4 | 0.394 |
| Triglyceride (mg/dl) | 119.5 (24 – 514) | 131.5 (46 – 764) | 0.216 |
| HDL (mg/dl) | 50.0 (29 – 145) | 47.5 (24 – 99) | 0.160 |
| LDL (mg/dl) | 100.9 ± 36.0 | 105.5 ± 35.0 | 0.519 |
| Creatinine (mg/dl) | 0.92 ± 0.18 | 0.90 ± 0.14 | 0.647 |
| CRP (mg/dl) | 0.05 (0.03 – 3.14) | 0.09 (0.03 – 1.12) | 0.145 |
| Apolipoprotein B (mg/dl) | 75.1 ± 22.2 | 81.4 ± 21.4 | 0.163 |
| Apolipoprotein A1 (mg/dl) | 112.8 ± 32.0 | 119.2 ± 22.9 | 0.266 |
| Lipoprotein(a) (mg/dl) | 16.7 ± 14.3 | 13.3 ± 11.6 | 0.203 |
| Medication |  |  |  |
| Calcium channel blocker | 49 (98.0) | 47 (94.0) | 0.617 |
| Vasodilators | 22 (44.0) | 24 (48.0) | 0.688 |
| Coronary angiography |  |  |  |
| Coronary stenosis**^*^** |  |  |  |
| No fixed stenosis | 21 (42.0) | 26 (52.0) | 0.316 |
| LAD stenosis | 22 (44.0) | 14 (28.0) | 0.096 |
| LAD %DS | 30 (20-50) | 30 (20-50) | 0.208 |
| LCX stenosis | 11 (22.0) | 8 (16.0) | 0.444 |
| LCX %DS | 40 (30-60) | 30 (20-50) | 0.367 |
| RCA stenosis | 5 (10.0) | 7 (14.0) | 0.538 |
| RCA %DS | 30 (20-60) | 30 (20-40) | 0.513 |
| Spasm location |  |  | 1.000 |
| Multivessel | 1 (2.0) | 1 (2.0) |  |
| LAD | 21 (42.0) | 21 (42.0) |  |
| LCX | 10 (20.0) | 11 (22.0) |  |
| RCA | 18 (36.0) | 17 (34.0) |  |
| Spontaneous spasm | 2 (4.0) | 1 (2.0) | 1.000 |
| Distal TIMI flow |  |  |  |
| TIMI 0 | 24 (48.0) | 33 (66.0) |  |
| TIMI ≥1 | 26 (52.0) | 17 (34.0) |  |

Data are presented as mean ± standard deviation, median (range), or n (%)

**^*^** Fixed stenosis was observed in 53 patients and 67 vessels were involved.

Abbreviations: CRP, C-reactive protein; HDL, high-density lipoprotein; LAD, left anterior descending artery; LCX, left circumflex artery; LDL, low-density lipoprotein; %DS, percent diameter stenosis; PCI, percutaneous coronary intervention; RCA, right coronary artery; TIMI, thrombolysis in myocardial infarction.

**Supplementary Table 2. Outcomes according to the characteristics of baseline coronary spasm**

|  | **Spontaneous spasm**  **(n = 3)** | **Distal TIMI**  **0 flow**  **(n = 56)** | **Distal TIMI**  **1 or 2 flow**  **(n = 41)** | **P value** |
| --- | --- | --- | --- | --- |
| Follow-up CAG^✝^ | N=3 | N=38 | N=21 |  |
| Coronary vasospasm |  |  |  |  |
| No change | 2 (66.7) | 15 (39.5) | 11 (52.4) | 0.501 |
| Remission | 1 (33.3) | 23 (60.5) | 10 (47.6) | 0.501 |
| Complete remission | 0 (0) | 6 (15.8) | 8 (38.1) | 0.095 |
| Progression | 0 (0) | 0 (0) | 0 (0) |  |

Data are presented as n (%).

^✝^62 patients underwent CAG for 1-year follow-up.

Abbreviations: CAG, coronary angiography.

**(2) Supplementary Figure and Figure Legends**

**Supplementary Figure 1. Study flow**


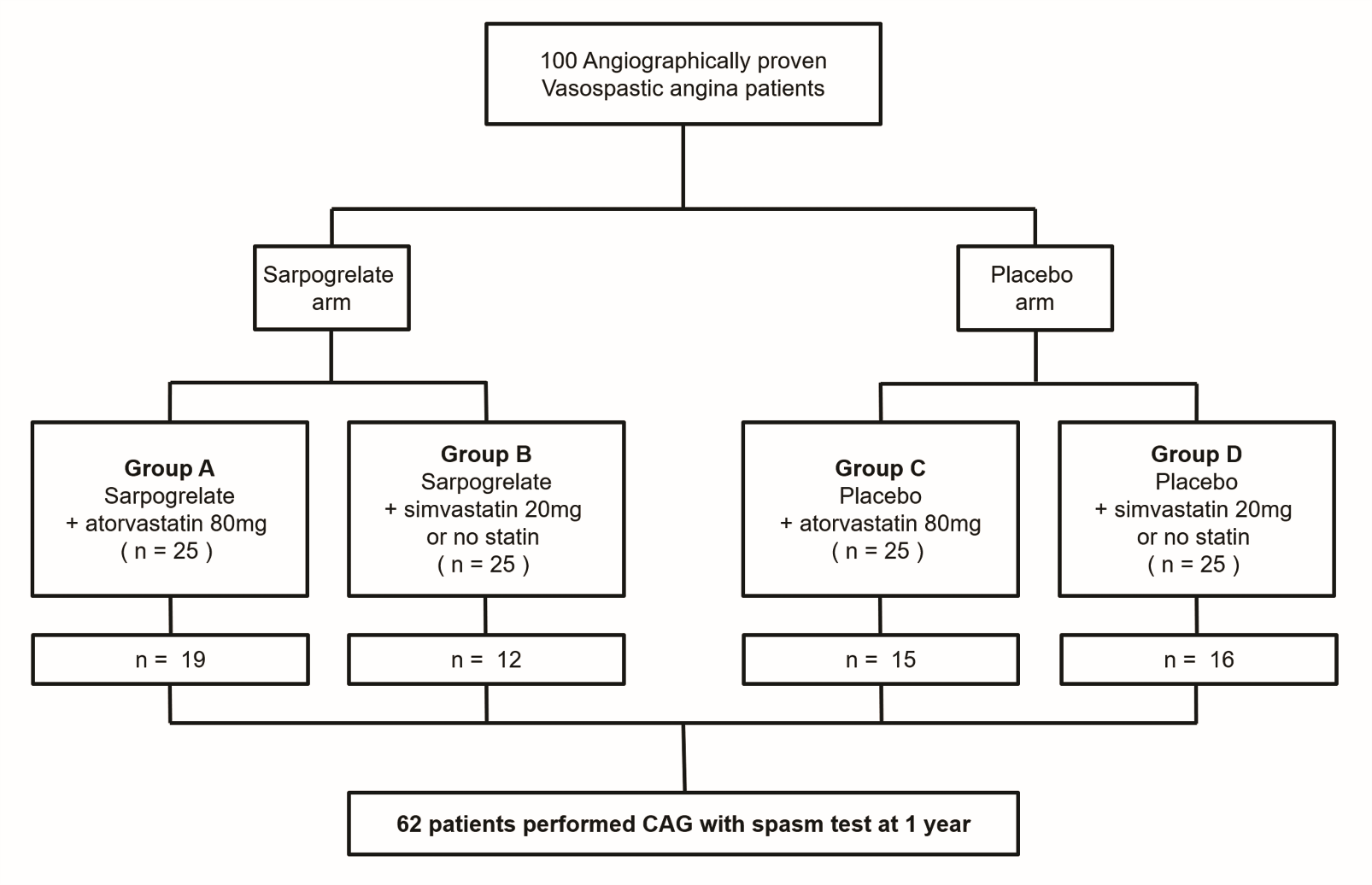


Abbreviations: CAG, coronary angiography

**Supplementary Figure 2. Compliance**


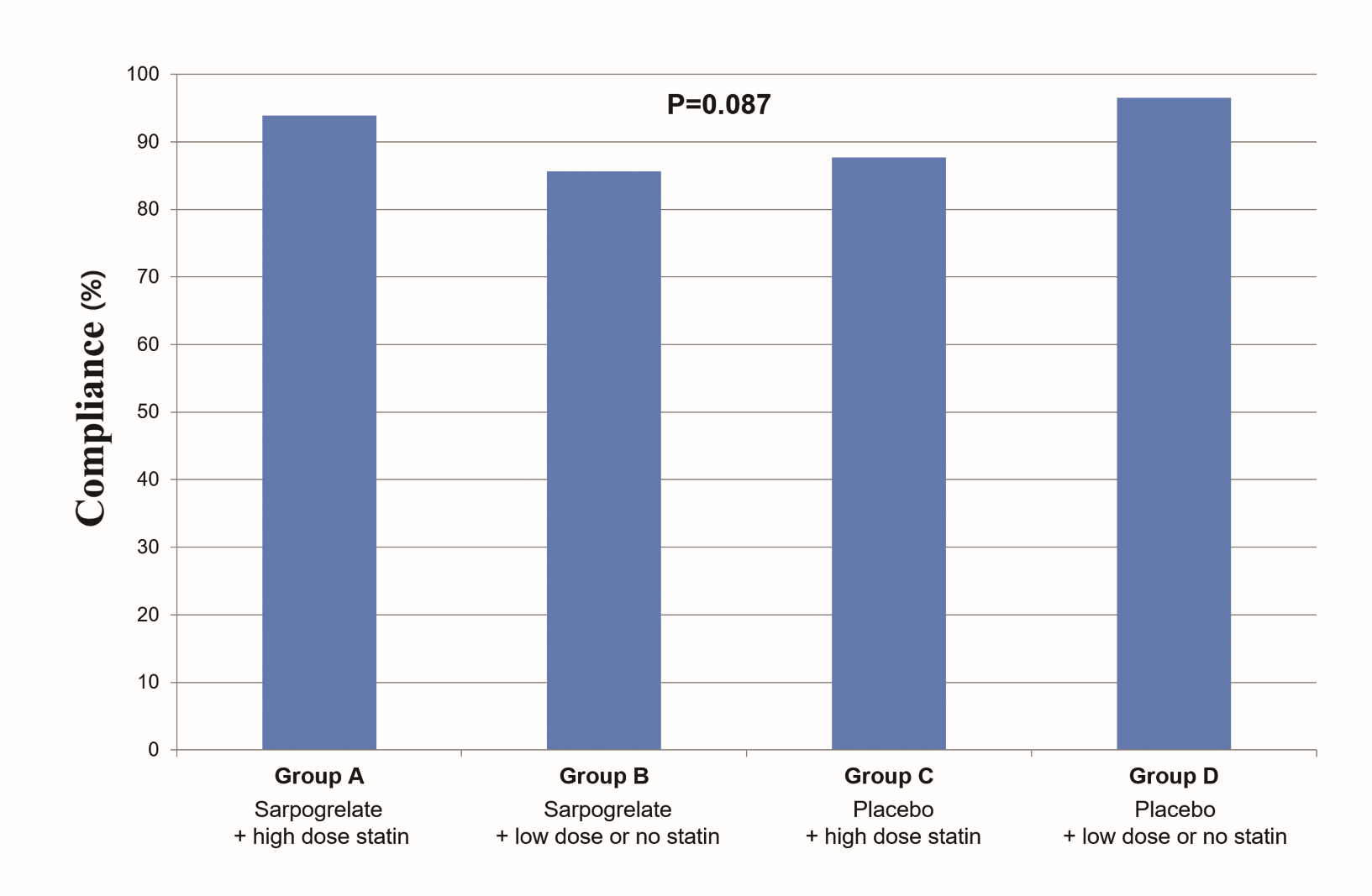


Bar graphs show the compliance of each group. Compliance is defined as the proportion of medication actually administered divided by the given tablets.
